# Histone acetyltransferase NAA40 modulates acetyl-CoA levels and lipid synthesis

**DOI:** 10.1186/s12915-021-01225-8

**Published:** 2022-01-20

**Authors:** Evelina Charidemou, Maria A. Tsiarli, Andria Theophanous, Vural Yilmaz, Chrysoula Pitsouli, Katerina Strati, Julian L. Griffin, Antonis Kirmizis

**Affiliations:** 1 Department of Biological Sciences, University of Cyprus, 2109 Nicosia, Cyprus; 2 Department of Biochemistry and Cambridge Systems Biology Centre, University of Cambridge, Cambridge, CB2 1GA UK; 3 Hammersmith Campus, UK Dementia Research Institute at Imperial College, Burlington Danes Building, Imperial College London, Du Cane Road, London, W12 0NN UK; 4 Section of Biomolecular Medicine, Department of Metabolism, Division of Systems Medicine, Digestion and Reproduction, The Sir Alexander Fleming Building, Exhibition Road, South Kensington, Imperial College London, London, SW7 2AZ UK

**Keywords:** Histone acetyltransferases, NAA40, acetyl-CoA, Lipid metabolism, Epigenetics, *Drosophila melanogaster*, Fat body, Metabolic disorders

## Abstract

**Background:**

Epigenetic regulation relies on the activity of enzymes that use sentinel metabolites as cofactors to modify DNA or histone proteins. Thus, fluctuations in cellular metabolite levels have been reported to affect chromatin modifications. However, whether epigenetic modifiers also affect the levels of these metabolites and thereby impinge on downstream metabolic pathways remains largely unknown. Here, we tested this notion by investigating the function of N-alpha-acetyltransferase 40 (NAA40), the enzyme responsible for N-terminal acetylation of histones H2A and H4, which has been previously implicated with metabolic-associated conditions such as age-dependent hepatic steatosis and calorie-restriction-mediated longevity.

**Results:**

Using metabolomic and lipidomic approaches, we found that depletion of NAA40 in murine hepatocytes leads to significant increase in intracellular acetyl-CoA levels, which associates with enhanced lipid synthesis demonstrated by upregulation in de novo lipogenesis genes as well as increased levels of diglycerides and triglycerides. Consistently, the increase in these lipid species coincide with the accumulation of cytoplasmic lipid droplets and impaired insulin signalling indicated by decreased glucose uptake. However, the effect of NAA40 on lipid droplet formation is independent of insulin. In addition, the induction in lipid synthesis is replicated in vivo in the *Drosophila melanogaster* larval fat body. Finally, supporting our results, we find a strong association of NAA40 expression with insulin sensitivity in obese patients.

**Conclusions:**

Overall, our findings demonstrate that NAA40 affects the levels of cellular acetyl-CoA, thereby impacting lipid synthesis and insulin signalling. This study reveals a novel path through which histone-modifying enzymes influence cellular metabolism with potential implications in metabolic disorders.

**Supplementary Information:**

The online version contains supplementary material available at 10.1186/s12915-021-01225-8.

## Background

Over the past decade, there have been numerous advances in understanding the dynamic relationship between metabolism and the epigenome. Metabolism is believed to be a principal regulator of the epigenome as chromatin-modifying enzymes are responsive to fluctuating central metabolite levels [1]. For instance, histone acetylases (HATs) and histone methyltransferases (HMTs) use acetyl coenzyme A (acetyl-CoA) and S-adenosyl methionine (SAM), respectively, as the sole cofactors for their catalytic reactions [2, 3]. Therefore, metabolite fluctuations can influence the abundance of certain histone modifications [4], which in turn may associate with specific transcriptional outputs or, as alternatively proposed, act as ‘sinks’ to maintain metabolite homeostasis [1, 5–8]. However, the opposite path of this reciprocal relationship between metabolism and chromatin-modifying enzymes is underexplored. Specifically, it remains largely elusive if chromatin-modifying enzymes influence the levels of specific metabolites and consequently impinge on cellular metabolism.

Acetyl-CoA is a central metabolic intermediate that acts as a key precursor for de novo lipogenesis (DNL) and as sole donor of acetyl groups for histone acetylation [9]. Therefore, DNL and histone acetylation compete for the same nucleocytosolic acetyl-CoA pool [10, 11]. DNL synthesises ‘new’ fat utilising cytosolic glycolysis-derived acetyl-CoA as a carbon source. During glycolysis, glucose generates pyruvate that is oxidised to citrate in mitochondria. Mitochondrial-derived citrate is exported to the cytosol where it is lysed by ATP-citrate lyase (ACL) to cytosolic acetyl-CoA. Similarly, histone acetylation in mammalian cells depends on ACL-mediated acetyl-CoA production [12], further suggesting that DNL and histone acetylation share the same precursor. Alternatively, acetate, a very short-chain fatty acid that can be derived from diet or from the deacetylation of histones, can be ligated with CoA to synthesise acetyl-CoA through acyl-CoA short-chain synthetase family member 2 (ACSS2). In fact, ACSS2 has been implicated in the utilisation of acetate as an alternative source of carbon to support lipid synthesis and histone acetylation under hypoxic conditions and in the absence of ACL [13, 14]. Interestingly, this intersection between DNL and histone acetylation is exemplified by the fact that decreasing acetyl-CoA flux towards the fatty acid synthesis pathway results in global increase of histone acetylation [11], and reprogramming of lipid metabolism provides a major acetyl-CoA source for histone acetylation [15]. However, it is unknown if conversely epigenetic enzymes could influence the intracellular levels of acetyl-CoA which would then preferentially rewire the DNL pathway. Such interplay between epigenetic enzymes and metabolism would have important clinical implications, since DNL has been linked to insulin resistance and nonalcoholic fatty liver disease [16].

N-alpha-acetyltransferase 40 (NAA40) is a highly conserved histone acetyltransferase that belongs to the N-terminal acetyltransferase (NAT) family of enzymes [17]. NAA40 selectively catalyses acetylation at the alpha-amino group of serine 1 on histones H4 and H2A [18, 19]. Recent studies have illuminated an important role for NAA40 in tumour growth control and metastasis in various types of cancers including lung, liver, and colorectal [20–24]. Additionally, previous reports have implicated this epigenetic enzyme in metabolic-associated conditions such as age-associated hepatic steatosis and calorie-restriction-mediated longevity [25, 26], but its link to cellular metabolism remains poorly characterised.

In this study, we set out to investigate the impact of NAA40 function on cellular metabolism. Using metabolomics and lipidomics, we show that depletion of NAA40 in murine hepatocytes leads to increased cellular acetyl-CoA levels and DNL induction manifested as enrichment in diglycerides and triglycerides. Τhese lipid changes culminate into an accumulation of cytoplasmic lipid droplets. Moreover, induction of lipid synthesis upon NAA40 depletion coincides with impaired insulin pathway and decreased glucose uptake. In addition, the increased lipid synthesis mediated upon NAA40 knockdown was validated in vivo in the *Drosophila* larval fat body, which is the functional equivalent of the mammalian liver and adipose tissue. In support of these observations, we also find an association of NAA40 expression with insulin sensitivity in obese patients. Overall, our findings demonstrate that loss of NAA40 acetyltransferase results in enhanced acetyl-CoA levels associating with increased fatty acid synthesis, and thus unveiling a novel path through which histone-modifying enzymes could impact cellular metabolism.

## Results

### Loss of NAA40 results in increased acetyl-CoA levels and induction of lipid synthesis genes in hepatocytes

**Fig. 1:**
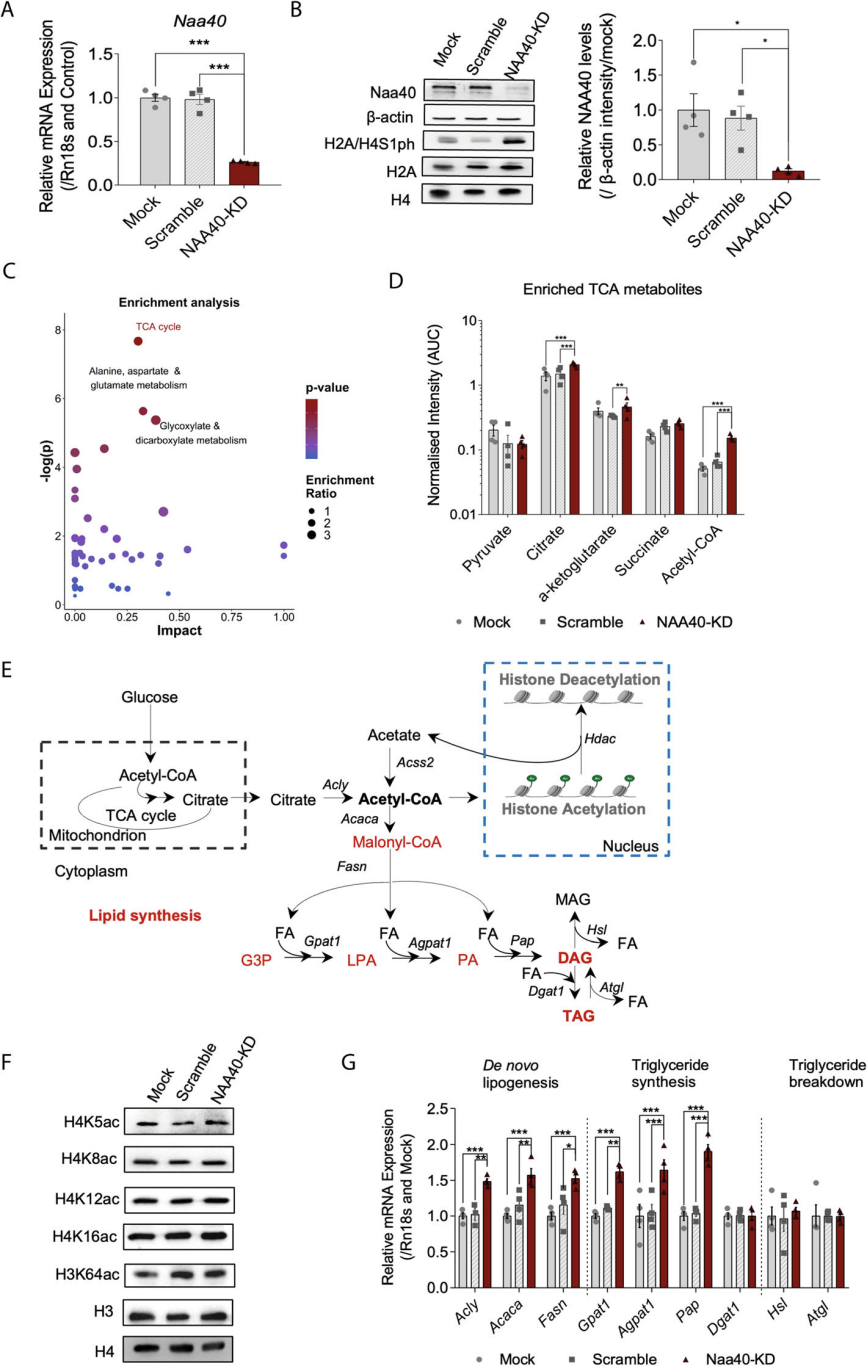
Metabolic reprogramming after NAA40 depletion in AML12 hepatocytes. **A** RT-qPCR analysis of expression of NAA40 mRNA levels in mock, scramble, and NAA40-KD cells after 48 h of siRNA treatment (*n* = 4/group). **B** Representative immunoblots (left) of protein extracts run in quadruplicate using antibodies against NAA40 and β-actin as loading control as well as western blot analysis of histone extracts using antibodies against the NAA40 antagonistic mark H2A/H4S1ph and H3, H2A, and H4 as loading controls in mock, scramble, and NAA40-KD cells after 48 h of siRNA treatment. Right plot indicates quantification of immunoblot signals NAA40 relative to β-actin. **C** Aqueous metabolites obtained by LC-MS 48 h after siRNA treatment were subjected to Metaboanalyst for enrichment and pathway analysis. **D** Analysis of the indicated TCA cycle intermediates in mock, scramble, and NAA40-KD cells measured by LC-MS after 48 h of siRNA treatment, with acetyl-CoA having the most significant changes (*n* = 4/group). **E** Schematic representation of the synthesis and consumption of cytosolic acetyl-CoA in mammalian cells. **F** Representative immunoblots of histone extracts run in triplicate using antibodies against the indicated histone acetylation marks and H3/H4 as loading controls in mock, scramble, and NAA40-KD cells after 48 h of siRNA treatment (*n* = 3/group). **G** RT-qPCR analysis of DNL synthesis genes *Acly*, *Acaca*, *Fasn*; triglyceride synthesis genes, *Gpat1*, *Agpat1*, *Pap* and Dgat1 and breakdown genes, *Hsl* and *Atgl* after 48 h of siRNA treatment (*n* = 4/group). All data are presented as mean ± SEM and analysed by 2-way ANOVA with post hoc Tukey’s multiple-comparisons test; *P ≤ 0.05, **P ≤ 0.01, ***P ≤ 0.001. Individual values can be found in Additional file 9: Table S3

The histone acetyltransferase NAA40 has been previously shown to be highly expressed in mouse liver [20] but also its protein and mRNA expression was readily detected in human liver among proteomic and transcriptomic studies, indicating that it might have an important biological function in the liver (Additional file 1: Fig. S1). In addition, the implication of NAA40 in hepatic steatosis and glucose sensing led us to hypothesise that this enzyme may impinge on cellular metabolism in the liver. To address this, we decided to perform metabolomics analysis in liver cells lacking NAA40. We initially depleted NAA40 using a pool of four different *Naa40* siRNAs which efficiently reduced its mRNA levels in AML12 hepatocytes compared to scramble and mock control cells (Fig. 1A). Accordingly, the protein levels of NAA40 were also robustly diminished after transfection with the NAA40 siRNAs compared to controls (Fig. 1B; quantification from three independent experiments in Additional file 2: Fig. S2). Furthermore, NAA40 depletion was validated by detection of its antagonistic histone phosphorylation mark at serine 1 of histones H2A and H4 (H2A/H4S1ph) [21], which was highly increased in the NAA40-KD cells, but not in control cells (Fig. 1B).

Following the robust depletion of NAA40 in AML12 cells, we next examined whether this affects the levels of metabolites. Notably, metabolomic analysis of NAA40-KD hepatocytes revealed significant enrichment of the TCA cycle pathway (Fig. 1C) and closer analysis showed a strong enrichment in citrate and acetyl-CoA levels in NAA40-KD hepatocytes compared to scramble and mock control cells (Fig. 1D). The accumulation of these TCA cycle metabolites suggests that NAA40-KD cells are in an anabolic state. To confirm this, we measured the ratio of the cellular redox NADH/ NAD+, which reflects the cellular energy balance. The reduced cofactor NADH is used for biosynthesis, whilst NAD+ has been found to inhibit synthesis and promote catabolism [27]. We found that upon NAA40 depletion NADH/NAD+ ratio significantly increased whilst the levels of ATP remain unchanged (Additional file 3: Fig. S3), reflecting an increase in the energy balance and not an oxidative catabolic state. The increase in the energy balance inhibits the flow of the TCA cycle resulting in the accumulation of citrate and acetyl-CoA to prime biosynthetic pathways [10, 28–30].

In light of this result, we wondered if this augmentation of citrate and acetyl-CoA levels in NAA40-depleted cells associates with stimulation of its major biosynthetic pathways (Fig. 1E). Therefore, we first examined if the levels of various histone acetylation marks were altered in NAA40-KD hepatocytes and detected no changes when compared to control cells (Fig. 1F). Thus, we next sought to determine whether the lipid synthesis pathway is affected upon NAA40 depletion and enhancement of acetyl-CoA levels. To address this, we first examined the expression of key genes involved in this pathway (Fig. 1E). Interestingly, we found that the relative expression of de novo lipogenesis (*Acly*, *Acaca*, *Fasn*) and triglyceride synthesis (*Gpat1*, *Agpat1*, *Pap*) genes increased in NAA40-KD cells compared to control cells, whilst the expression of triglyceride breakdown genes (*Hsl*, *Atgl*) remained unchanged (Fig. 1G). Overall, these data demonstrate that diminishing NAA40 in cells results in increased intracellular acetyl-CoA levels which coincide with increased expression of lipid synthesis genes but not with alterations in histone acetylation levels.

### NAA40 depletion in hepatocytes enhances lipid synthesis

**Fig. 2:**
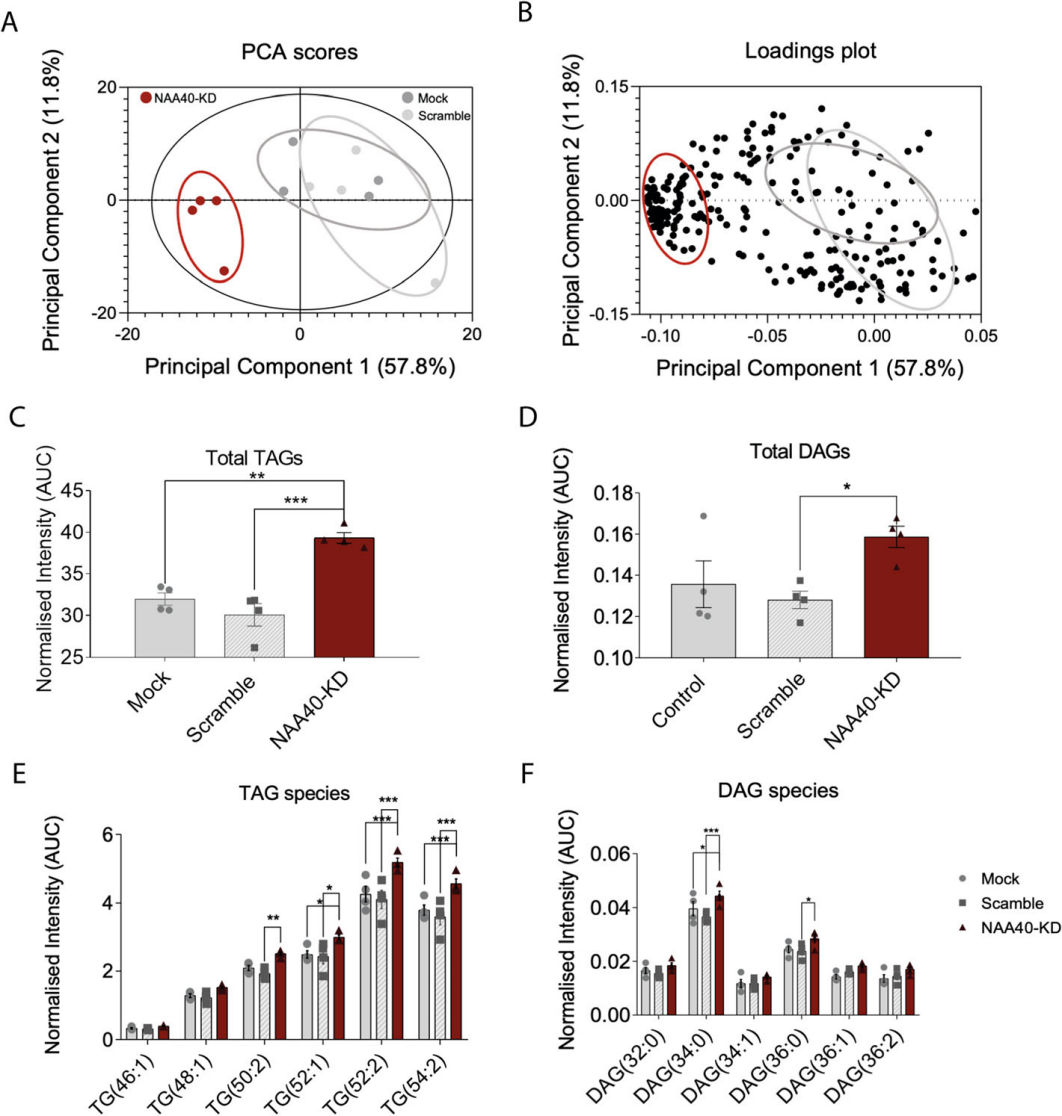
NAA40 knockdown increases intact lipid content affecting specific DAG and TAG species. **A** PCA scores plot of the total lipid profiles in mock, scramble, and NAA40-KD cells after 48 h of siRNA treatment. Dark grey circles represent independent mock samples, light grey represent scramble samples and red circles represent NAA40-KD samples (*n* = 4/group). **B** PCA loadings plot showing the lipid species influence on the separation between the mock, scramble, and NAA40-KD sample groups (*n* = 4/group). **C** Sum of TAG content (*n* = 4/group), **D s**um of DAG content and (*n* = 4/group). **E** Intracellular levels of short-chain TAG (*n* = 4/group) and **F** DAG species measured by LC-MS after 48 h of siRNA treatment (*n* = 4/group). All data are presented as mean ± SEM and analysed by 1-way ANOVA with post hoc Dunnett’s multiple-comparisons test; *P ≤ 0.05, ** *P* ≤ 0.01, *** *P* ≤ 0.001. Individual values can be found in Additional file 9: Table S4

The previous findings suggested that diminishing NAA40 might induce lipid synthesis, and hence, we sought to examine the lipid profile of NAA40-depleted hepatocytes. The lipidome of NAA40-KD, as well as scramble and mock control hepatocytes, was analysed using HPLC-MS. Resulting data were processed by principal component analysis (PCA) to identify the distribution of lipids between NAA40-KD, scramble, and mock hepatocytes. PCA indicated that the distribution of the four NAA40-KD replicate samples was well-discriminated, in terms of lipid composition, compared to the scramble and mock negative control samples, which grouped together (Fig. 2A). The loadings plot revealed that diglycerides and triglycerides represent the major discriminant variables within the NAA40-KD sample group (Fig. 2B). Consistently, focused analysis of triglycerides and diglycerides showed that their total levels were increased upon NAA40 depletion compared to control cells (Fig. 2C, D, respectively). Particularly, amongst the most discriminant triglycerides (TAG) and diglycerides (DAG) species driving the separation between the NAA40-KD sample group and the negative control groups were TAG 50:2, TAG 52:1, TAG 52:2, TAG 54:2, DAG 34:0, DAG 36:0, and DAG 36:1 (Fig. 2E, F) that have been previously described as lipid markers of metabolic risk [31, 32]. Notably, the observed lipid enrichment was consistent with the changes in the expression of genes described above, which control DAG and TAG synthesis, but not their breakdown (Fig. 1E, G). Altogether these findings suggest that the increased levels of acetyl-CoA upon loss of NAA40 associate with stimulation of fatty acid synthesis.

### NAA40 knockdown attenuates the insulin pathway and decreases glucose uptake

**Fig. 3:**
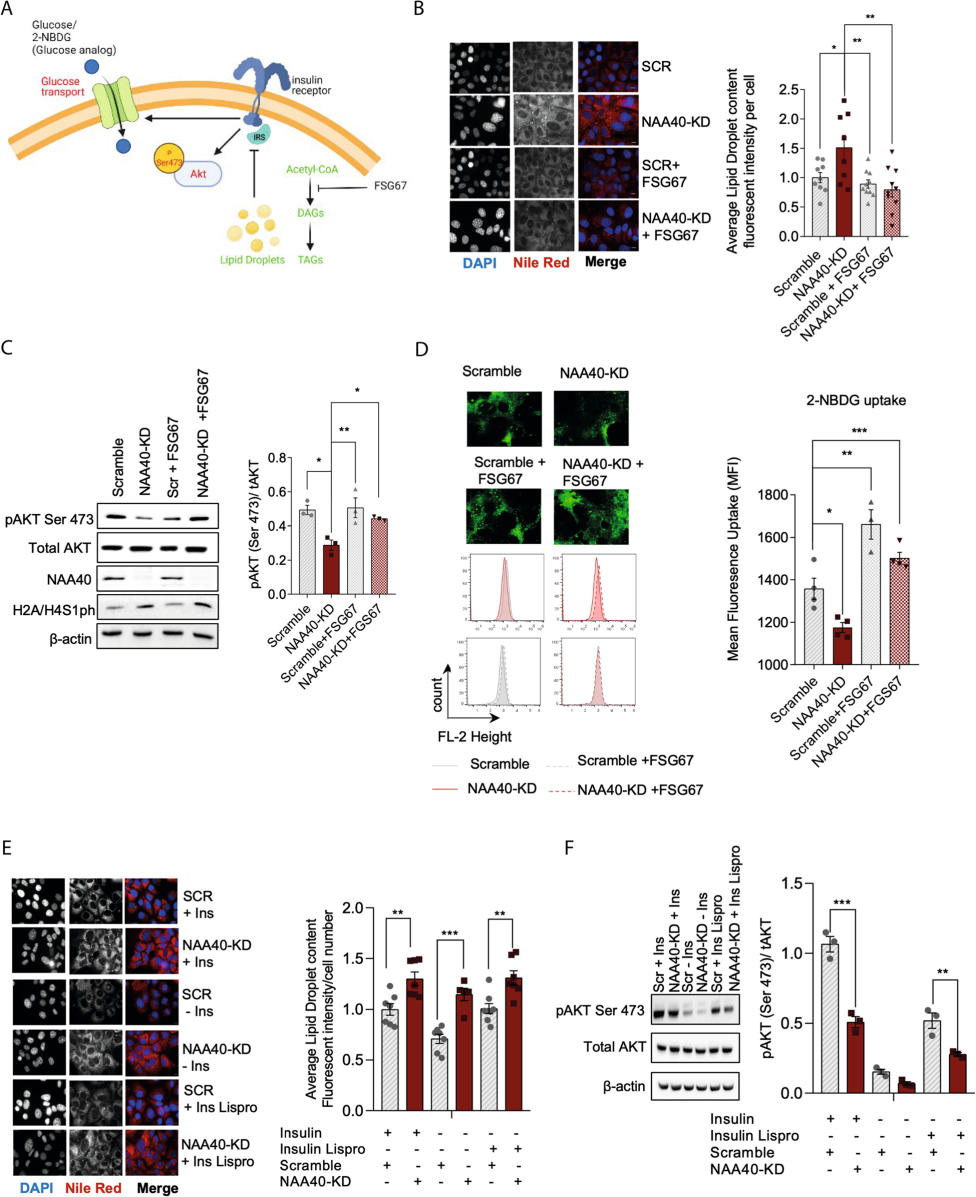
NAA40 affects accumulation of lipid droplets, the insulin pathway, and glucose uptake. **A** Schematic representation of acquired insulin resistance from accumulated lipid droplets. Illustration was created with BioRender.com. **B** Representative image of lipid droplets by Nile red (red) and nuclei by DAPI (blue) in scramble and NAA40-KD cells with and without FSG67, 48 h after siRNA treatment; Scale bar = 25 μm. Right plot represents the quantification of Nile red using ImageJ (*n* = 8–10/group). **C** Representative immunoblot (right) run in triplicate of cell extracts using antibodies against pAKTser473, total AKT, and β-actin as loading control in scramble and NAA40-KD cells with and without FSG67, 48 h after siRNA treatment. Right plot indicates quantification of immunoblot signals pAKT-ser473 relative to total AKT (*n* = 3/group). **D** Representative images (top) and histograms (bottom) show comparative glucose uptake between scramble and NAA40-KD cells with and without FSG67, after 48 h of siRNA treatment; cells were incubated with 2-NBDG in glucose free media for 30 min. Right plot shows mean fluorescence uptake quantification in scramble and NAA40-KD cells with and without FSG67, after 48 h of siRNA treatment (*n* = 4/group). **E** Representative image of lipid droplets by Nile red (red) and nuclei by DAPI (blue) in scramble and NAA40-KD cells with and without insulin or insulin lispro, 48 h after siRNA treatment; Scale bar = 25 μm. Right plot represents the quantification of Nile red using ImageJ (*n* = 7–10/group). **F** Representative immunoblot (right) run in triplicate of cell extracts using antibodies against pAKTser473, total AKT, and β-actin as loading control in scramble and NAA40-KD cells with and without insulin or insulin lispro, 48 h after siRNA treatment. Right plot indicates quantification of immunoblot signals pAKT-ser473 relative to total AKT (*n* = 3/group). Data are presented as mean ± SEM and analysed by 1-way ANOVA with post hoc Dunnett’s multiple-comparisons test; *P ≤ 0.05, **P ≤ 0.01, ***P ≤ 0.001. Individual values can be found in Additional file 9: Table S5

The neutral lipid species described above are normally stored in the cytoplasm in the form of lipid droplets. Increased lipid droplet content is sufficient to activate protein kinase Cε (PKCε), which in turn inhibits the kinase activity of the insulin receptor substrate (IRS), thereby inducing insulin resistance [33]. This impaired IRS kinase activity is typically reflected by inactivation of insulin cascade intermediates, such as dephosphorylation of AKT serine 473 (pAKT Ser473), that is normally phosphorylated upon insulin stimulation [Fig. 3A, [33]]. Accordingly, NAA40*-*depleted cells had significantly increased content of lipid droplets (Fig. 3B) and decreased levels of pAKT Ser473 compared to negative control cells (Fig. 3C), consistent with the enhancement of lipid synthesis observed above in NAA40-KD cells.

To support the notion that the enhanced acetyl-CoA levels upon loss of NAA40 are indeed responsible for lipid droplet accumulation, we inhibited histone deacetylation using sodium butyrate which is a potent histone deacetylase (HDAC) inhibitor [34]. HDACs generate acetate that can be ligated to CoA to synthesise acetyl-CoA through ACSS2; therefore, their inhibition can drive acetyl-CoA towards the histone acetylation path rather than lipid synthesis (Additional file 4: Fig. S4A). Remarkably, inhibition of histone deacetylation increased the levels of specific histone acetylation marks (Additional file 4: Fig. S4B) that were previously unchanged in NAA40-KD cells (Fig. 1F), and significantly reduced lipid droplet accumulation (Additional file 4: Fig. S4C). This suggested acetyl-CoA was shuffled to histone acetylation rather than being used as a substrate for lipid synthesis but also that HDAC-derived acetate is an important source for lipids upon NAA40 depletion. Indeed, silencing of NAA40 significantly increased the expression of the ACSS2 gene that is needed for the synthesis of acetyl-CoA from acetate (Additional file 4: Fig. S4D). In addition, double silencing of NAA40 and ACSS2 diminished lipid droplet formation (Additional file 4: Fig. S4E), recapitulating the effect of HDAC inhibition, reflecting that acetate is an important source of NAA40-KD-depedent lipid synthesis.

To further confirm the effect of NAA40 on lipid metabolism, we inhibited the lipid synthesis pathway using the Glycerol-3-phosphate acyltransferase 1 (GPAT1) inhibitor FSG67 [35]. When NAA40-KD hepatocytes were treated with FSG67, we no longer detected accumulation of lipid droplets compared to untreated NAA40-KD cells, which resembled the lipid droplet content of scramble control cells (Fig. 3B). Consistently, the levels of pAKT Ser473 in NAA40-KD hepatocytes treated with FSG67 were restored almost back to the levels of control cells (Fig. 3C).

Since decreased pAKT Ser473 suggests that the insulin pathway is impaired upon NAA40 depletion, we examined glucose uptake using the fluorescent glucose analogue 2-NBDG. As predicted, flow cytometry analysis indicated that glucose uptake was significantly decreased in NAA40-KD hepatocytes compared to negative control cells (Fig. 3D). Importantly, glucose uptake was restored in NAA40-KD cells when treated with the lipid synthesis inhibitor FSG67 (Fig. 3D), confirming that NAA40-KD impacts glucose uptake through its effect on lipid synthesis.

To evaluate the role of NAA40 depletion on lipid droplet formation and insulin signalling, hepatocytes were transfected with siRNA for 48 h in the presence or absence of insulin or its analogue, insulin lispro [36]. Even though the absence of insulin decreased the amount of lipid droplets in control cells as expected, it did not affect the accumulation of lipid droplets upon NAA40 depletion (Fig. 3E). In agreement, the levels of pAKT Ser473 upon NAA40 depletion were decreased compared to corresponding scramble controls in the presence or absence of insulin and insulin lispro (Fig. 3F). These data suggest that lipid droplet accumulation upon NAA40 depletion is independent of the presence of insulin and this accumulation can subsequently impact the activation of the insulin signalling cascade.

Taken together, these data indicate that lack of NAA40 increases acetyl-CoA levels leading to neutral lipid accumulation, in the form of lipid droplets that impair the insulin signalling cascade ultimately decreasing glucose uptake.

### Metabolic rewiring upon NAA40 depletion precedes potential transcriptional effects of NAA40

**Fig. 4:**
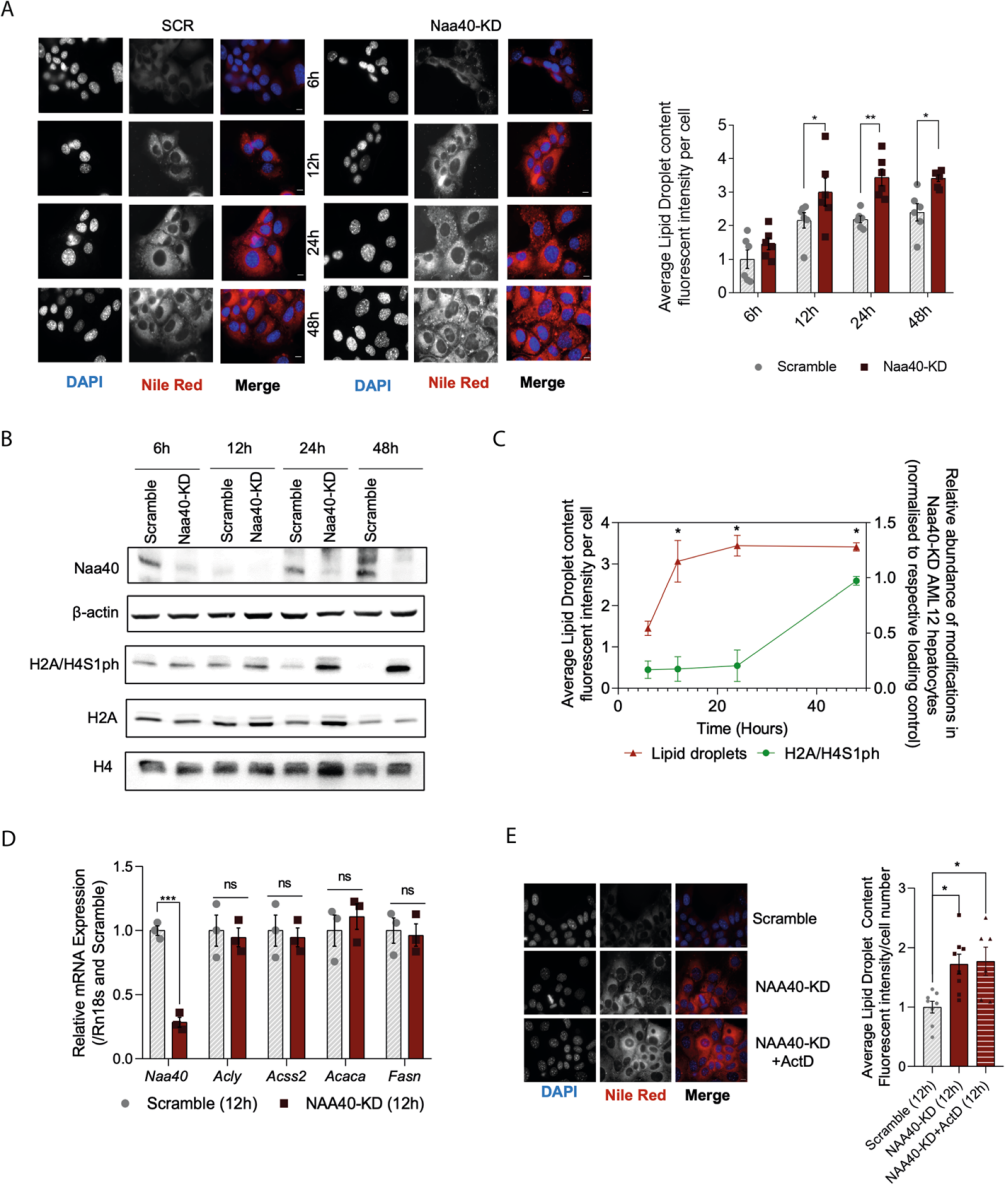
Metabolic rewiring upon NAA40 depletion precedes potential transcriptional effects of NAA40. **A** Visualisation (right) and quantification (left) of neutral lipids by Nile red (red) and nuclei by DAPI (blue) in scramble and NAA40-KD cells, 6, 12, 24, and 48 h of siRNA treatment; Scale bar = 25 μm (*n* = 6/group). **B** Representative immunoblot of whole cell extracts run in triplicate using antibodies against NAA40 and β-actin, as well as western blot analysis of histone extracts using antibodies against the indicated histones and H2A/H4S1 phosphorylation mark (*n* = 3/group). **C** Relative abundance of H2A/H4S1ph measured by western blots as well as of lipid droplets measured by Nile red staining 6, 12, 24, and 48 h of siRNA treatment. **D **RT-qPCR analysis of *Naa40* and DNL synthesis genes *Acly*, *Acss2*, *Acaca*, and *Fasn* after 12 h of siRNA treatment (*n* = 3/group)**. E** Visualisation (right) and quantification (left) of neutral lipids by Nile red (red) and nuclei by DAPI (blue) in scramble, NAA40-KD and NAA40-KD + Actinomycin D cells for 12 h; Scale bar = 25 μm (*n* = 6–8/group). Data are presented as mean ± SEM and analysed by 1-way ANOVA with post hoc Dunnett’s multiple-comparisons test; *P ≤ 0.05, **P ≤ 0.01, ***P ≤ 0.001. Individual values can be found in Additional file 9: Table S6

As a histone-modifying enzyme, knockdown of NAA40 may affect the expression of a plethora of genes, including metabolic genes that might be responsible for the observed induction in lipid synthesis. To address this scenario, we first examined the temporal kinetics of lipid droplet formation and the currently known transcriptional effect of NAA40 through its crosstalk with the histone mark H2A/H4S1ph [21, 37]. We found that lipid droplet accumulation (at 12 h) occurred prior to the increase of the NAA40-antagonistic histone mark H2A/H4S1ph, which only started to significantly rise from 24 h onwards (Fig. 4A–C). In addition, at 12 h where there is a significant accumulation of lipid droplets, the expression of DNL genes was not altered (Fig. 4D) but only at a later time point (at 48 h; Fig. 1E and Additional file 4: Fig. S4D). Lastly, transcriptional inhibition through actinomycin D treatment [38] for 12 h did not abolish lipid droplet formation upon NAA40 depletion (Fig. 4E), indicating that at this timepoint newly synthesised transcripts are not required for the observed rewiring in lipid metabolism. These results suggest that metabolic rewiring upon NAA40 depletion precedes potential transcriptional deregulation by NAA40, suggesting that NAA40 might impact lipid metabolism directly through influencing acetyl-CoA levels. In addition to our data, this notion is also supported by evidence in literature suggesting that initial changes in acetyl-CoA levels can subsequently influence the expression of DNL genes [39].

### Fat body-specific depletion of NAA40 in *Drosophila* induces lipid synthesis

**Fig. 5:**
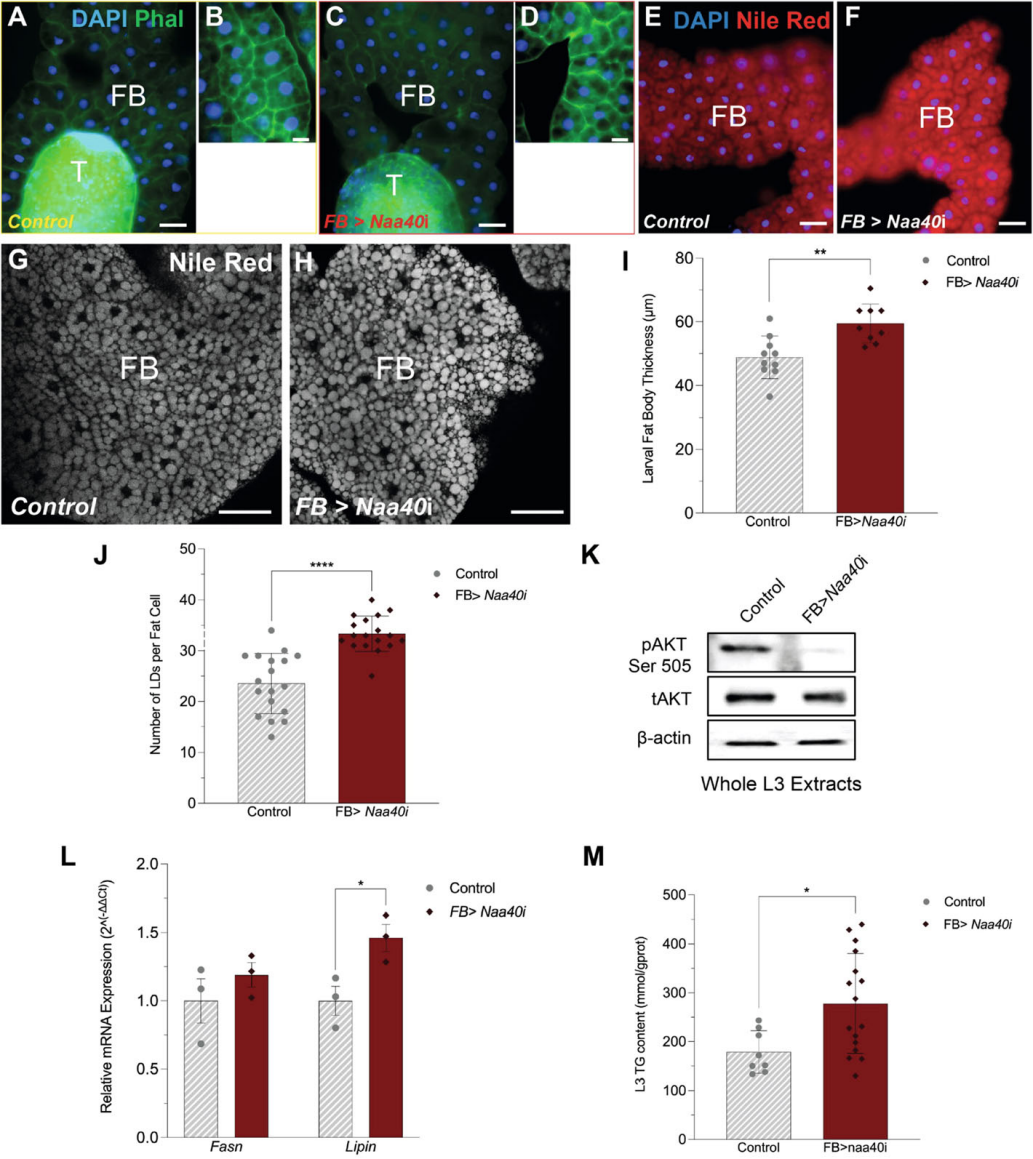
NAA40 depletion in *Drosophila* larval fat body promotes lipogenesis and triglyceride accumulation. FB-specific Naa40 knockdown was achieved with the use of the FB-specific GAL4 promoter fly line, 0.68 Lsp2-GAL4, crossed to a UAS-Naa40RNAi line (FB > Naa40i). **A–D** Phalloidin staining of L3 FBs. Panels A and B outlined in yellow represent widefield images taken at × 20 and × 40 respectively of a control FB. Panels **C** and **D** outlined in red, are corresponding × 20 and × 40 images from a Naa40RNAi FB. **E, F** Widefield images of Nile red-stained FBs acquired at the same settings as in **A–D**. **G, H** Confocal imaging of Nile red-stained FBs. Optical stacks (3 optical sections × 0.5 μm each) of control and Naa40RNAi-stained FBs, obtained at the same settings. Scale bars **A–F** 200 μm and **C, D** 50 μm (**I**) FB thickness measurements of control and Naa40RNAi samples (ncontrol = 10; nFB > Naa40i = 9). **J** Number of lipid droplets (LDs) per fat cell in control and Naa40RNAi samples (LDs in a total of six fat cells were evaluated for three different biological samples per group). **K** Representative immunoblot run in triplicate of protein extracts prepared from whole L3 larvae for pAKTSer505, tAKT, β-actin in control, and Naa40RNAi conditions (*n* = 3/group). **L** RT-qPCR analysis of Lipin and Fasn in control and Naa40RNAi FBs (*n* = 3/group). **M** Triglyceride (TG) concentration (mmol/g protein) in whole L3 larvae (ncontrol = 8; nFB > Naa40i = 16). All data are presented as mean ± SEM and analysed by unpaired 2-tailed Student’s *t* test; *P ≤ 0.05, **P ≤ 0.01, ***P ≤ 0.001. Individual values can be found in Additional file 9: Table S7

We found that lack of NAA40 enhances lipid synthesis in vitro in hepatocytes. To determine if loss of NAA40 has similar effects in vivo, we used the *Drosophila melanogaster* (fruit fly) fat body (FB) as a model. The fly FB is an organ analogous to the mammalian liver and fat tissue [40]. We performed FB-specific knockdown (KD) of NAA40 using the GAL4-UAS system [41, 42], which resulted in significant reduction of *Naa40* expression in the third instar (L3) larval FB (Additional file 5: Fig. S5). We then explored the effects of *Naa40*-KD on FB development and lipid content during the L3 larval stage. Phalloidin staining showed that FBs depleted of *Naa40* (*Naa40*^RNAi^) had similar fat cell architecture compared to control FBs (Fig. 5A–D). However, Nile red staining of neutral lipids displayed higher lipid content in *Naa40*^RNAi^ compared to control FBs, as indicated by the more intense staining through conventional fluorescence microscopy (Fig. 5E, F). To validate this observation, we performed confocal imaging of Nile red-stained FBs (Fig. 5G, H) and confirmed the higher intensity in *Naa40*^RNAi^ FBs compared to control FBs imaged at the same settings (Fig. 5G). The enhanced lipid staining was further supported by the increased FB thickness observed in *Naa40*^RNAi^ compared to control FBs (Fig. 5I). Furthermore, NAA40 silencing led to increased numbers of lipid droplets per fat cell compared to control cells (Fig. 4 J), similar to our observations in AML12 hepatocytes (Figs. 3B and 4A). Moreover, FB-specific NAA40 knockdown results in decreased phosphorylation of the *Drosophila* lipid synthesis effector kinase AKTSer505 (Fig. 5 K), which is equivalent to the murine pAKTSer473 [43] that was similarly decreased in the NAA40-KD hepatocytes (Fig. 3C). Lastly, we observed upregulation of *lipin*, a gene central in lipid metabolism and orthologous to mammalian *lipin1/pap* [44], in *Naa40*^RNAi^ compared to control FBs (Fig. 5 L). To further evaluate the impact of Naa40 deletion on lipid metabolism, we quantified the total levels of triglycerides (TGs) in L3 larvae, since TG are the main component of fat. This analysis revealed increase in TG levels following *Naa40*^RNAi^ (Fig. 5 M), in agreement with the other results on FB architecture and LD content. Overall, these findings demonstrate that in vivo depletion of *Naa40* enhances lipid production both on the transcriptional and molecular level (TG), in addition to affecting insulin signalling effectors such as AKT, as observed in AML12 hepatocytes.

### NAA40 expression in the liver of obese patients associates with insulin sensitivity

**Fig. 6:**
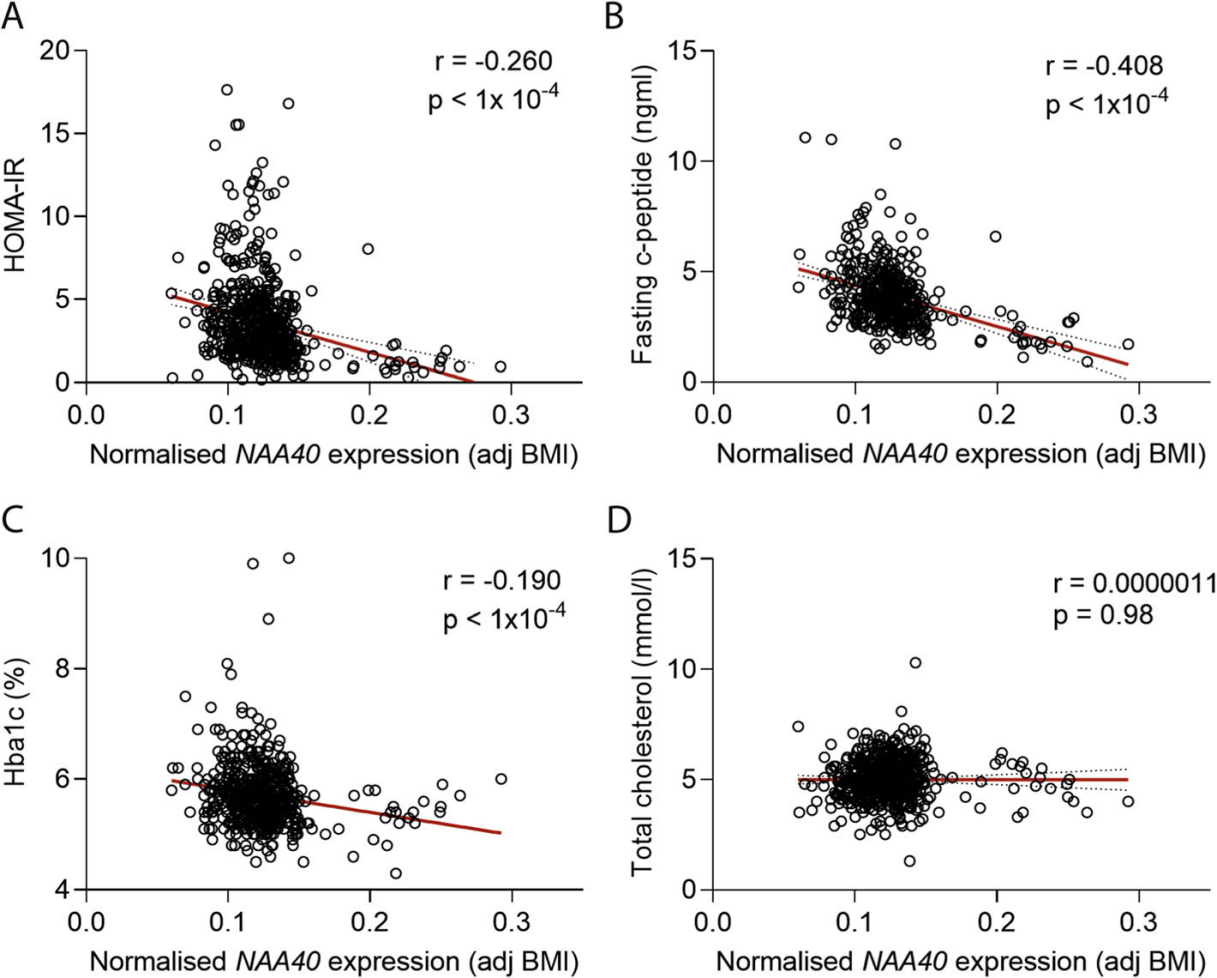
NAA40 expression inversely correlates with insulin-resistant traits in obese patients. **A** Scatter plots illustrating the correlation between the expression of NAA40 (adj BMI) and HOMA-IR index, **B** fasting c-peptide levels, **C** Hbac (%), and **D** cholesterol in 575 obese patients; samples extracted from GEO database; GSE130991. The red line indicates the linear regression slope. Statistical analysis was performed using Pearson’s rank correlation coefficient (*r*)

The alteration in lipid droplet content of hepatocytes and fly FBs described above, as well as its effects on the insulin pathway suggest that NAA40 levels may be implicated in insulin resistance. To explore this possibility, we examined the association of NAA40 expression and insulin resistance in patients with metabolic disease. Specifically, we analysed publicly available hepatic transcriptome and clinical data from the Biological Atlas of Severe Obesity (ABOS) cohort (GEO; GSE130991). From these, we excluded statin- and diabetes-treated patients whose lipid profile might be affected. In this cohort (*n* = 576), we calculated insulin resistance using the homeostatic model assessment for insulin resistance (HOMA-IR) by taking into account fasting glucose and insulin concentration [45]. Analysis of NAA40 expression in the liver of these patients showed a significant negative correlation between NAA40 mRNA levels and HOMA-IR index (*r* = − 0.206, *p* < 1 × 10^−4^) (Fig. 6A). In addition, the expression levels of NAA40 negatively correlated with other insulin-resistant traits such as c-peptide levels (*r* = − 0.408, *p* < 1 × 10^−4^) and Hba1c (%) (*r* = − 0.190; *p* < 1 × 10^−4^) (Fig. 6B, C, respectively). In contrast, the expression of NAA40 did not correlate with total cholesterol levels in the liver of obese patients (Fig. 6D). Overall, this analysis indicates that NAA40 levels in the human liver might be associated with defects in insulin signalling.

## Discussion

Metabolic regulation of histone post-translational modifications and its biological significance has recently emerged through various studies [1, 4, 46, 47]. This significant body of work has focused on how fluctuations of metabolite levels influence the deposition and removal of chromatin modifications. However, the role of histone-modifying enzymes in controlling the levels of specific intracellular metabolites and possible downstream effects on metabolic pathways has remained largely unexplored [1, 5, 6]. Since the highly conserved histone N-terminal acetyltransferase NAA40 has been previously linked with metabolic conditions [24–26], in the present study we investigate its impact on metabolite levels and subsequent metabolic perturbations. We show that NAA40 deficiency results in metabolic rewiring by increasing acetyl-CoA levels and inducing lipid droplet formation. In turn, the altered metabolic dynamics upon NAA40 loss lead to impaired insulin signalling thereby decreasing glucose uptake. Our findings provide a novel connection between epigenetic enzymes and metabolic regulation with implications in metabolic syndrome.

A number of studies have been focused on glucose and fructose as ‘lipogenic’ nutrients that contribute to the acetyl-CoA pool [48]. In fact, high-carbohydrate and high-fructose diets increase the rate of de novo lipogenesis by loading the system with a large substrate pool [49, 50]. More recently, it has been shown that elevated branched chain amino acids (BCAAs) contribute to 25% of lipogenic acetyl-CoA [51]. Interestingly, our work provides an alternative mechanism of control, through epigenetic modifiers, leading to the generation of lipogenic acetyl-CoA. More specifically, disrupting NAA40 function increases the levels of acetyl-CoA, which importantly does not alter the histone acetylation status of the cell, suggesting that the excess acetyl-CoA is shuffled into lipid synthesis (Fig. 1). This notion is also consistent with the induction of genes involved in de novo lipogenesis (Fig. 1). Furthermore, this is corroborated by the fact that inhibition of histone deacetylases resulted in enhanced histone acetylation, possibly by fuelling additional acetyl-CoA towards this path, and thus decreasing lipid accumulation in NAA40-depleted cells (Additional file 4: Fig. S4).

It is well established that in liver cells insulin binds to its receptor and activates sequentially the insulin receptor substrate-1 (IRS-1), 1-phosphatidylinositol 3-kinase (PI3K), and AKT to enable GLUT4 translocation to the plasma membrane, ultimately allowing glucose transport [52]. Accordingly, our data demonstrate that NAA40 depletion attenuated this defined insulin signalling (Fig. 3). First, loss of NAA40 significantly reduced phosphorylation of AKT S473 in its hydrophobic motif, which is typically a mark of activated insulin signalling [53, 54]. Second, cells lacking NAA40 show decreased glucose uptake, which is the final step affected when the insulin cascade is impaired. Third, NAA40 knockdown results in aberrant accumulation of lipid droplets in hepatocytes which has already been implicated in a mechanism leading to impaired insulin signalling [55, 56]. Specifically, we observed lipid changes in TAG 50:2, 52:2, 54:2, and DAG 34:0 (Fig. 2) that have been previously linked to DNL-mediated steatosis, insulin resistance, and cardiovascular disease [31, 32, 57–59]. Finally, inhibition of lipid synthesis in NAA40-depleted cells rescued both phosphorylation of AKT and glucose uptake (Fig. 3), effects that are consistent with restoration of insulin signalling. Notably, defective insulin regulation of glucose transport represents the hallmark of insulin resistance, which is an early pathological feature of type 2 diabetes [60].

The link of NAA40 to lipid biosynthesis is further supported by our in vivo work, using the *Drosophila melanogaster* FB model system. The fly FB and oenocytes are functionally analogous to the human liver [61–63], and most disease genes, including metabolic disease-causing genes, are conserved between humans and flies [63]. Consistent with our mammalian in vitro results, FB-specific knockdown of *Naa40* leads to lipid droplet accumulation, increased FB thickness and upregulation of de novo lipid synthesis genes such as *fasn* and *lipin* (Fig. 5A–L)*.* Moreover, these changes are accompanied by increase in triglyceride levels (Fig. 5M). Interestingly, Aregger et al*.* recently performed a screen to identify genetic interactions implicated in de novo fatty acid synthesis and revealed that NAA40 genetically interacts with FASN [64], further supporting the implication of NAA40 within this metabolic pathway. Collectively, these results underscore a conserved role for NAA40 in metabolic rewiring and lipid metabolism. Given the fact that *Drosophila* is a powerful genetic model, it could be exploited in future research to further dissect the mechanism behind the role of NAA40 in metabolic rewiring.

NAA40 is thought to acetylate histones co-translationally during their production in S-phase of the cell cycle, and therefore, the abovementioned effect on lipid biosynthesis and droplet formation might depend on an active cell cycle. A recent report demonstrates that even though NAA40 expression is increased during S phase, arresting colorectal cancer cells in the G0/G1 phase of the cell cycle does not affect the metabolic rewiring induced upon NAA40 depletion, reflecting that the effects of NAA40 depletion are independent of an active cell cycle [37].

Although depletion of NAA40 leads to significant increase in acetyl-CoA levels, it remains unclear how this is achieved. We envisage two different scenarios on how NAA40 might control acetyl-CoA levels. The first involves NAA40-mediated transcriptional regulation of genes controlling acetyl-CoA production. This is supported by a previous observation from our group, which demonstrated that, in yeast cells, NAA40 controls the expression of glycolysis-related genes [25]. Therefore, it is possible that loss of NAA40 de-represses a transcriptional programme, which stimulates acetyl-CoA production. In the second, more intriguing and favourable scenario, we speculate that loss of the NAA40 results in decreased consumption of acetyl-CoA by this acetyltransferase thereby leading to an accumulation of this metabolite within cells. This second scenario is supported by the fact that NAA40 acetylates approximately 92–98% of cellular histones H4 and H2A [18, 65–67], which is an estimated acetyl-CoA consumption of around 92–98 μmol/L and corresponds to almost five times the amount of free intracellular acetyl-CoA (20 μmol/L) levels (Additional file 6: Table S1). This makes NAA40 a major consumer of acetyl-CoA within the cell and therefore, disrupting NAA40 function might significantly alter the availability of this key metabolite. Interestingly, within this study, various evidence suggest that metabolic rewiring upon NAA40 depletion precedes potential transcriptional deregulation by NAA40. Specifically, the observed increase in de novo synthesis of lipids upon NAA40-depletion (at 12 h) preceded the enhancement of the NAA40-antagonising histone mark H2A/H4S1ph (Fig. 4A–C), which has been previously linked to NAA40-mediated transcriptional effects [21, 37]. In addition, the expression of DNL genes does not change at 12 h, when lipid accumulation is observed, and transcriptional inhibition at this time point did not abolish lipid droplet formation. This evidence argues that acetyl-CoA accumulation might occur prior to chromatin changes upon loss of NAA40. This suggests that reduced consumption, and not NAA40-mediated transcriptional regulation, drives the build-up of acetyl-CoA. Future studies are needed to determine if this is a general mechanism for other HATs and whether the acetyl-CoA consumption level of different HATs will have different impacts on the metabolic phenotype in vitro and in vivo.

## Conclusions

Taken together, our findings reveal a role for NAA40 in regulating lipid metabolism and insulin signalling. We demonstrate that depleting NAA40 increases acetyl-CoA levels and this coincides with increased lipid synthesis and decreased insulin sensitivity. Importantly, these results provide insight for a new link between epigenetic dysregulation and metabolic syndrome.

## Methods

### Cell culture

AML 12 cells were purchased from American Type Culture Collection (ATCC, RRID: CVCL_0140), and they were tested negative for mycoplasma contamination. AML12 were cultured in 1:1 (v/v) Dulbecco’s modified Eagle’s medium and Ham’s F12 medium (Thermo), supplemented with 10% foetal bovine serum (FBS), 1% penicillin/streptomycin (100 units/mL and 100 μg/mL, respectively), 1% insulin-transferrin-selenium (ITS; 10 mg/L, 5.5 mg/L and 6.7 μg/L respectively), and dexamethasone (100 μmol/L) at 37 °C in 5% CO_2_. Cells were plated at a density of 50,000 cells/well in collagen 1-coated 12-well plates (Cat. # 7340295, Corning) and grown to confluence in maintenance medium.

### siRNA-mediated knockdown

*Naa40*, *Acss2*, or *NAA40 + Accs2* in AML 12 cells were silenced by siRNA transfection (Horizon Discovery). In brief, cells were plated at a density of 100,000 cells/well and on day 2, cells were transfected with 25 nM siRNA specific for *Naa40* (QIAgen; cat. # GS70999) or *Acss2* (QIAgen; cat. # GS60525) or negative control (QIAgen; cat. #1027281) or water (mock) using DharmaFECT 1 Transfection Reagent (Horizon discovery) according to the manufacturer’s instructions. Cells were harvested at 6, 12, 24, and 48 h after transfection.

### Insulin treatments

Transfected cells were treated in presence or absence of insulin (10 mg/L; Gibco cat. #41400045) or the insulin analogue insulin lispro (10 mg/L; Sigma, cat. #1342321).

### Acetylation inhibition

Transfected cells were treated with 5 mM sodium butyrate (Sigma, Cat. B5887), for 48 h.

### Lipid synthesis inhibition

Transfected cells were treated with 30 μM FSG67, a GPAT inhibitor (Focus Biomolecule, Cat. 10-4577), for 48 h.

### Transcription inhibition

Transfected cells were treated with 1 μg/ml of Actinomycin D, a class II gene transcription inhibitor (Santa Cruz, Cat. # CAS 50-76-0.), for 24 h.

### Metabolite extractions

Metabolites and lipids were extracted from cells using a modified method of Folch and colleagues [68]. Briefly, pelleted cells were homogenised in chloroform/methanol (2:1, v/v, 750 μL); including a mixture of deuterated internal standards. Samples were sonicated for 15 min and deionised water was added (300 μL). The organic (upper layer) and aqueous (lower layer) phases were separated following centrifugation at 13,000×*g* for 20 min. The organic phase extracts containing lipids were dried under a stream of nitrogen gas whilst the aqueous samples were dried in a CentriVap Centrifugal Concentrator with attached cold trap (78100 series, Labconco Co, Kansas City, USA).

### Analysis of aqueous metabolites by triple quadrupole mass spectrometry

Aqueous extracts were reconstituted in acetonitrile: 10 mM ammonium carbonate (7:3, v/v, 50 μL) containing an internal standard mix (AMP ^13^C_10_, ^15^N_5_; ATP ^13^C_10_, ^15^N_5_; Glutamate U^13^C, U^15^N; Leucine-d10, Phenylalanine-d5, Proline U^13^C, U^15^N; and Valine-d8). Samples were injected onto a Vanquish UHPLC attached to a TSQ Quantiva triple quadrupole mass spectrometer (Thermo Scientific) with a heated ESI source. Samples were analysed with normal phase and reverse phase chromatography.

For the normal phase analysis, metabolites were separated with a BEH-amide (150 × 2.1 mm 1.7 μm) column at 30 °C. The mobile phase consisted of (A) 0.1% of ammonium carbonate and (B) acetonitrile and was pumped at a flow rate of 0.6 mL/min. The gradient was programmed as follows: 80% of B for 1.50 min followed by a linear decrease from 80 to 40% of B for 3.5 min and finally returned to initial conditions.

For reverse phase analysis, samples were dried and reconstituted in 10 mM ammonium acetate solution and analysed with an ACE C18 PFP (150 × 2.1 mm 5 μm) column at 30 °C. The mobile phase consisted of (A) 0.1 % formic acid in water and (B) 0.1 % formic acid in acetonitrile, pumped at 0.5 mL/min. The gradient was programmed as follows: 0% of B for 1.60 min followed by a linear increase from 0 to 30% of B for 4 min and to 90% by 4.5 min, held for 1 min and then returned to initial conditions.

The mass spectrometer was operated in SRM mode; collision energies and RF lens voltages were generated for each species using the TSQ Quantiva optimisation function. Xcalibur Software (Thermo Scientific) was used to identify peaks, process mass spectra, and normalise data to the closest-eluting internal standard.

### Analysis of intact lipids using high-resolution mass spectrometry

To the organic fraction an internal standard mix was incorporated [N-palmitoyl-d_31_-D-erythro-sphingosine (16:0-d_31_ Ceramide), pentadecanoic-d_29_ acid (15:0-d_29_ FFA), heptadecanoic-d_33_ acid (17:0-d_33_ FFA), eicosanoic-d_39_ acid (20:0-d_39_ FFA), 1-palmitoyl(D_31_)-2-oleyl-sn-glycero-3-phosphatidylcholine (16:0-d_31_-18:1 PC), 1-palmitoyl(d_31_)-2-oleyl-sn-glycero-3-phosphoethanolamine (16:0-d_31_-18:1 PE), 1-palmitoyl-d_31_-2-oleoyl-sn-glycero-3-[phospho-rac-(1-glycerol)] (16:0-d_31_-18:1 PG), N-palmitoyl(d_31_)-d-erythro-sphingosylphosphorylcholine (16:0-d_31_ SM), glyceryl tri(pentadecanoate-d_29_) (45:0-d_87_ TAG), glyceryl tri(hexadecanoate-d_31_) (48:0-d_93_ TAG) (Avanti Polar Lipids Inc, USA)] and dried down under nitrogen. The organic fraction was reconstituted in 100 μL chloroform/methanol (1:1, v/v), and 10 μL of the resulting solution added to 90 μL isopropanol (IPA)/acetonitrile (ACN)/water (2:1:1, v/v). Analysis of the fractions was performed using an LTQ Orbitrap Elite Mass Spectrometer (Thermo Scientific, Hemel Hempstead, UK).

In positive mode, 5 μL of sample were injected onto a C18 CSH column, 1.7 μM pore size, 2.1 mm × 50 mm (Cat # 186005296, Waters Ltd, Manchester, UK) which was held at 55 °C in a Dionex Ultimate 3000 ultra-high performance liquid chromatography system (UHPLC; Thermo Scientific). A gradient (flow rate 0.5 mL/min) of mobile phase A (ACN/water 60:40, 10 mmol/L ammonium formate) and B (LC–MS-grade ACN/IPA 10:90, 10 mmol/L ammonium formate) was used. In negative ion mode, 10 μL of the sample was injected and 10 mmol/L ammonium acetate was used as the additive to aid ionisation.

In both positive and negative ion mode, the gradient began at 40% B, increased to 43% B at 0.8 min, 50% B at 0.9 min, 54% B at 4.8 min, 70% B at 4.9 min, 81% B at 5.8 min, peaked at 99% B at 8 min for 0.5 min, and subsequently returned to the starting conditions for another 1.5 min to re-equilibrate the column. The UHPLC was coupled to an electrospray ionisation (ESI) source which ionised the analytes before entering the mass spectrometer. Data were collected in both positive and negative ion mode with a mass range of 110–2000 *m/z*. Default instrument-generated optimisation parameters were used.

The spectra files were converted to mzML format and features picked using xcms [69], after retention time alignment. Lipid identification was performed using an in-house R script. Peak areas of each metabolite were normalised to the appropriate internal standard and tissue weight.

### Glucose assay uptake

A 2-deoxy-2-[(7-nitro-2,1,3-benzoxadiazol-4-yl) amino]-D-glucose (2-NBDG) glucose uptake assay was performed according to the manufacturer’s instructions (Biovision Milpitas, CA). In brief, after starving cells for 6 h with 0.5% FBS-containing DMEM, 2-NBDG was added for 30 min at 37 °C in 5% CO_2_. Cells were harvested with trypsin and centrifugation at 1200 rpm for 5 min. The pellet was resuspended in PBS and the fluorescent uptake was measured using flow cytometry. Data were acquired on a Bio-Rad S3e Cell Sorter and analysed using FlowJo software (Treestar).

### Fly stocks and crosses

Fly stocks used in this study are *w*^*1118*^ (BDSC# 3605, RRID: BDSC_3605)*; 0.68-lsp2 Gal4* [kindly provided from Brigitte Dauwalder, University of Houston] [70] and *UAS-Naa40*^*RNAi*^ (VDRC# KK101213) [71]. Transgenic RNAi fly stocks were obtained from the Vienna *Drosophila* Resource Center (VDRC, www.vdrc.at). For FB-specific *Naa40* KD, *0.68-lsp2* GAL4 virgin females were crossed to *UAS-Naa40*^*RNAi*^ males, whereas, for the control conditions, GAL4 virgin females were crossed to *w*^*1118*^ males. All flies and crosses were maintained in 25 °C under a 12-h light/dark circle and crosses were allowed to proceed only for 1–2 days to avoid larval crowding. For our studies, we chose to use only male L3 progeny except the TG analysis were both male and female larvae were used.

### Gene expression analysis

Total RNA was extracted and purified from hepatocytes using a RNeasy Mini Kit (QIAgen) according to the manufacturer’s specifications. Purified RNA concentration was quantified at 260 nm using a NanoDrop 100 (Thermo Fisher Scientific). Third instar larval fat body total RNA (8-10 fat bodies per sample) was extracted using Trizol via guanidinium thiocyanate-phenol-chloroform using TRIzol™ Reagent (Thermo Fischer Scientific).

Each purified RNA sample was diluted with RNase-free water to a final concentration of 100 ng/μL. Complementary DNA (cDNA) synthesis and genomic DNA elimination in RNA samples was performed using an RT2 First Strand Synthesis kit (QIAgen) according to the manufacturer’s specifications. The reactions were stored at − 20 °C prior to real-time quantitative PCR analysis. The relative abundance of transcripts of interest was measured by qPCR in KAPA SYBR Green (SYBR Green Fast qPCR Master Mix) with a the Bio-Rad CFX96 detection system (Applied Biosystems). The SYBR Green qPCR Mastermix contained HotStart DNA Taq Polymerase, PCR Buffer, dNTP mix (dATP, dCTP, dGTP, dTTP), and SYBR Green dye. Before adding cDNA to each well of the 96-well plate, cDNA was diluted in RNase-free water to a final concentration of 8 ng/μL. PCR component mix was prepared by mixing 10 μL SYBR Green qPCR Mastermix with 0.6 μL of 10 μmol/L target primers (forward and reverse; 6 pmoles/reaction) and 4.4 μL RNase-free water. To each well of a 96-well plate, 5 μL cDNA (total amount 40 ng) and 15 μL PCR components mix were added. The plate was centrifuged at 1000×*g* for 30 s to ensure that the contents were mixed and to remove any bubbles present in the wells. The plate was placed in the real-time cycler with the following cycling conditions: 10 min at 95 °C for 1 cycle to activate HotStart DNA Taq Polymerase; 15 s at 95 °C and 1 min at 60 °C to perform elongation and cooling for 40 cycles. Sequences for qPCR Primers used for mouse *Rn18s*, *Fasn*, *Acaca*, *Gpat1*, *Agpat1*, *Pap*, *Dgat1*, *Hsl*, and *Atgl* and *Drosophila melanogaster actin*, *naa40*, *fasn* and *lipin* were purchased from Integrated DNA Technologies (Additional file 7: Table S2). Expression levels were normalised to the endogenous control, *Rn18s* for mouse *or actin* for drosophila, using the ΔΔCt method and fold changes reported were relative to the control group in the dose response (mock).

### Histone extracts

Histones were purified using the method of Shechter et al. [72].

### Triglyceride measurement

Sample processing for triglyceride quantification was performed as described in Werthebach et al. (2019) [73]. Briefly, three to six crawling L3 larvae were transferred in a 1.5-mL Eppendorf tube and homogenised in 100 μL 0.05% Tween20 in H_2_O per larva, using a motorised homogeniser. Then, the homogenate was heat inactivated by incubation at 70 °C for 5 min and centrifuged at 5000 rpm for 5 min. The supernatant was collected in a new tube and centrifuged at 14,800 rpm for 15 min at 4 °C. Triglyceride measurement was performed using the Triglyceride (TG) Colorimetric Assay Kit (Elabscience, USA) according to the manufacturer’s instructions. Protein values used to normalise TG measurements were quantified using the Bradford method. Both male and female larvae samples were collected and following comparison of TG levels with no sex-specific changes found, male and female measurements were pooled.

### Protein lysates

Cell pellets and *Drosophila* larvae (3–5 whole third instar larvae/replicate) were lysed in 100 μL Cell Extraction Buffer (10 mmol/L Tris, 100 mmol/L NaCl, 1 mmol/L EDTA, 1 mmol/L EGTA, 10 mmol/L NaF, 20 mmol/L Na_4_P_2_O_7_, 20 mmol/L Na_3_VO_4_, 1% Triton X-100, 10% glycerol, 0.1% sodium dodecyl sulfate, 0.5% deoxycholate, 1 mmol/L phenylmethylsulfonyl fluoride, complete protease inhibitor tablet, and 1% of each phosphatase cocktail inhibitor 2 and 3) for 30 min, vortexing at 10-min intervals. The lysate was centrifuged at 13,000×*g* for 10 min at 4 °C and the supernatant was collected and stored at − 80 °C.

### Western blotting

The protein concentration was measured by Bradford assay (Bio-Rad). Approximately 20–50 μg of protein was separated on SDS-PAGE and subsequently transferred to a nitrocellulose membrane (GE Healthcare). The membranes were blocked with 5% TBS-T/BSA for 1 h at room temperature and then incubated with the primary antibodies at 4 °C overnight. The primary antibodies used were as follows: NAA40 (1:1000, ab106408, Abcam, RRID: AB_10866699), H2AS1Ph/ H4S1PHh (1:1000, ab177309, Abcam, RRID: AB_2797594), phospho-Akt (Ser473) (1:1000, cat. 9271, Cell signalling, RRID: AB_329825), Akt (pan) (1:000, cat. C67E7, Cell Signaling, RRID: AB_915783), H4K5ac (0.5:25,000; cat. ab51997, Abcam, RRID: AB_2264109), H4K8ac (1:1000; cat. ab15823, Abcam, RRID: AB_880455), H4K12ac (1:5000; cat. A b46983, Abcam, RRID: AB_873859), H4K16ac (1:1000; cat. ab109463, Abcam, RRID: AB_10858987), H3K64ac (1:1000; cat. ab214808, Abcam, RRID: AB_2894996), H4 (1:1000; cat. 05-858, Millipore, RRID: AB_390138), H3 (1:000, ab1791, Abcam, RRID: AB_302613) and β-actin (1:1000; sc-1616-R, Santa Cruz, RRID: AB_630836). The membranes were incubated with the secondary antibody, horseradish peroxide (HRP)-conjugated goat anti-rabbit IgG (1:30,000, Scientific). Bands were visualised by the enhanced chemiluminescence system (BioRad) and analysed using densitometry using ImageJ analysis software (NJH).

Original immunoblot images can be found in Additional file 8: Fig S6.

### Lipid staining in AML12

Cells were seeded on coverslips in 12-well plates. Cells were washed with 1× PBS and fixed with 4% paraformaldehyde. Intracellular lipids were stained with Nile red (Invitrogen) as described by the manufacturer. Nuclei/DNA were stained with DAPI (Dako). Images were obtained using a Zeiss Axiovert 200 M microscope.

### Nile red (NR) staining of larval fat body and imaging

Fat bodies (FBs) from crawling third instar (L3) larvae were dissected in PBS and then fixed in 4% formaldehyde in PBS, for 30 min at room temperature. Following PBS rinses, FBs were incubated for 1 h in NR solution, in the dark. Then, excess NR was rinsed with water and samples were mounted on microscopy slides for imaging using 75% glycerol. Samples were imaged using Leica TCS SP2 AOBS DMIRE2 inverted scanning confocal microscope and images were visualised using ImageJ.

FB thickness was calculated by addition of the number of optical sections required to complete a full z-stack, with the top and bottom sections representing the immediately following section before no sample was visible. The number of LDs per fat cell were quantified in Adobe Photoshop CC 2017 with the count tool, using a modified version of Fan et al. [74]. Specifically, the number of LDs in 10 fat cells, from at least 3 biological samples were counted.

### Phalloidin staining

FBs were fixed in 4% formaldehyde for 20 min at room temperature and then washed with 0.2% PBS in Triton (0.2% PBSTr). Following 1 h blocking with 10% heat inactivated goat serum in 0.2% PBSTr, FBs were incubated with Alexa 488 Phalloidin (Molecular Probes, 1:500) in block for 2 h at room temperature. Following a brief rinse, samples were incubated with DAPI (1 μg/ul) for 10 min at room temperature. Then samples were washed with PBS and mounted on microscopy slides with Vectashield and imaged on a Zeiss Axioscope A.1 fluorescent microscope.

### Statistics

Multivariate statistical analyses were performed in Metaboanalyst 4.0 (www.metaboanalyst.ca). All variables were log transformed and subjected to principal component analysis (PCA) and pathway analysis and metabolite set enrichment analysis of significant metabolites. The extent to which the model fits and predicts the data is represented by *R*^2^ X and *Q*^2^ X, respectively.

Data were visualised using GraphPad (GraphPad Prism 8.0; GraphPad Software, San Diego, CA, USA). All data are expressed as means ± SEM. In GraphPad, unpaired *t*-test, one- or two-way ANOVA was performed where appropriate to determine significant differences between experimental groups. For a single comparison between two groups, an unpaired 2-tailed *t*-test was applied. For a single comparison between more than two groups, one-way ANOVA was used, whilst for two comparisons between more than two groups two-way ANOVA was used. For one-way ANOVA, Tukey’s post hoc multiple comparison test was performed, whilst for two-way ANOVA, Sidak’s post hoc multiple comparison test was used. Differences between experimental groups were statistically significant when *p* ≤ 0.05. All experiments were reproduced at least three times.

## Supplementary Information

**Additional file 1: Figure S1.** NAA40 is readily expressed in human liver amongst different transcriptomic and proteomic studies. Data were extracted from Human Expression Atlas of EMBL-EBI and were filtered to select only the high expression levels of NAA40 in RNA-sequencing and proteomic experiments. Each dot corresponds to the presence of NAA40, while where there is no dot, they are no available data.

**Additional file 2: Figure S2.** Silencing of Naa40 in AML12 Hepatocytes. **(A)** RT-qPCR analysis of expression of Naa40 mRNA levels in scramble and Naa40-KD cells after 48 h of siRNA treatment (*n* = 3/group). **(B)** Representative immunoblot run in triplicate of whole-cell extracts using antibodies against NAA40 and β-actin as loading control (*n* = 3/group). Data are presented as mean ± SEM and analysed by unpaired 2-tailed student’s t-test; *P ≤ 0.05, ** *P* ≤ 0.01, *** *P* ≤ 0.001. Individual values can be found in Additional file 9: Table S8.

**Additional file 3: Figure S3.** AML12 hepatocytes are in an anabolic state upon NAA40 depletion. Normalised intensity of NAD+, NADH, NADH/NAD+ and ATP measured by LC-MS in scramble and Naa40-KD cells after 48 h of siRNA treatment (*n* = 4/group). Data are presented as mean ± SEM and analysed by unpaired 2-tailed student’s t-test; *P ≤ 0.05, ** *P* ≤ 0.01, *** *P* ≤ 0.001. Individual values can be found in Additional file 9: Table S9.

**Additional file 4: Figure S4.** Histone deacetylation-derived acetate is an important source for lipid accumulation upon NAA40 depletion. **(A)** Schematic representation of the different routes for the synthesis of acetyl-CoA in mammalian cells. **(B)** Representative immunoblot run in triplicate using antibodies against the indicated histone acetylation marks in scramble and NA40-KD cells with and without sodium butyrate, 48 h after siRNA treatment (*n* = 3/group). **(C)** Visualisation (left) and quantification (right) of lipid droplets by Nile red (red) and nuclei by DAPI (blue) in scramble and NAA40-KD cells with and without sodium butyrate, 48 h after siRNA treatment; Scale bar = 25 μm (n = 6-10/group). **(D)** RT-qPCR analysis of expression of *Naa40*, *Acly and Acss2* mRNA levels in scramble and NAA40-KD, ACSS22-KD and NAA40-KD + ACSS2-KD cells after 48 h of siRNA treatment (*n* = 3/group)**. (E)** Visualisation (left) and quantification (right) of lipid droplets by Nile red (red) and nuclei by DAPI (blue) in NAA40-KD, ACSS22-KD and NAA40-KD + ACSS2-KD cells after 48 h of siRNA treatment; Scale bar = 25 μm (*n* = 6-8/group). Data are presented as mean ± SEM and analysed by 1-way ANOVA with post hoc Dunnett’s multiple-comparisons test; *P ≤ 0.05, ** *P* ≤ 0.01, *** *P* ≤ 0.001**.** Individual values can be found in Additional file 9: Table S10.

**Additional file 5: Figure S5**. Validation of NAA40 knock-down in L3 larvae fat bodies. RT-qPCR analysis of *Naa40* in control and NAA40-KD FBs (*n* = 3/group; 4-5 larvae FB in each biological repeat). Data are presented as mean ± SEM and analysed by unpaired 2-tailed student’s t-test; *P ≤ 0.05, ** *P* ≤ 0.01, *** *P* ≤ 0.001. Individual values can be found in Additional file 9: Table S11.

**Additional file 6: Table S1**. Estimated consumption of Acetyl-CoA by NAA40.

**Additional file 7: Table S2.** List of primer sequences used in the study.

**Additional file 8: Figure S6.** Uncropped blots.

**Additional file 9: Table S3-S11.** Raw data for graphs with N < 6.

**Additional file 10: Table S12.** Aqueous metabolites dataset. **Table S13**. Lipidomic dataset.

## Data Availability

All data generated or analysed during this study are included in this published article and its additional files. All uncropped blots can be found in Additional file 8. Raw data for experiments with *N* < 6 can be found in Additional file 9: Table S3-S11. The aqueous metabolomics and the lipidomic datasets can be found in Additional file 10: Table S12 and S13, respectively. The human dataset analysed during the current study is available in the GEO repository, https://www.ncbi.nlm.nih.gov/geo/query/acc.cgi?acc=GSE130991 [75].

## Abbreviations

Acetyl-CoA: Acetyl coenzyme A
ACL: ATP-Citrate Lyase
ACSS2: Acyl-CoA short-chain synthetase family member 2
BCAA: Branch chain amino acids
DAG: Diglycerides
DNL: De novo lipogenesis
FB: Fat body
GPAT1: Glycerol-3-phosphate acyltransferase 1
HATs: Histone acetylases/acetyltransferases
HDAC: Histone deacetylase
HMTs: Histone methylases/methyltransferases
HOMA-IR: Homeostatic Model Assessment for Insulin Resistance
IRS-1: Insulin receptor substrate-1
NAA40: N-alpha-acetyltransferase 40
NAT: N-terminal acetyltransferase
PCA: Principal component analysis
PI3K: 1-phosphatidylinositol 3-kinase
PKCε: Protein kinase Cε
SAM: S-adenosyl methionine
TAG: Triglycerides
